# General practitioners’ perceptions of their practice of evidence-based chronic disease prevention interventions: a quantitative study in Shanghai, China

**DOI:** 10.1186/s12875-020-01212-y

**Published:** 2020-07-22

**Authors:** Feng Fan, Zhaoxin Wang, Dehua Yu, Chen Chen, Delei Shen, Zhaohu Yu, Xin Liu, Huining Zhou, Jianwei Shi

**Affiliations:** 1 Yangpu Hospital affiliated to Tongji University School of Medicine, Shanghai, 200090 China; 2 Academic Department of General Practice, Tongji University School of Medicine, Shanghai, 200090 China; 3 School of Public Health, Shanghai Jiaotong University School of Medicine, Shanghai, 200025 China; 4 General Practice Center, Nanhai Hospital, Southern Medical University, Foshan, 528244 China; 5 Shanghai General Practice and Community Health Development Research Center, Shanghai, 200090 China; 6 Shanghai Jing’an District Jiangning Road Community Health Service Center, Shanghai, 200041 China; 7 Shanghai Fenglin Community Health Service Center, Shanghai, 200000 China; 8 Navy 971 Hospital, Qingdao, 266071 China

**Keywords:** General practitioner, Evidence-based, Implementation, China

## Abstract

**Background:**

Epidemic chronic diseases pose significant challenges to the improvement of healthcare in China and worldwide. Despite increasing international calls for the inclusion of evidence-based decision-making (EBDM) processes in chronic disease prevention and control programming as well as policymaking, there is relatively little research that assesses the current capacity of physicians and the factors that influence that capacity in China.

**Method:**

This cross-sectional study was conducted in community health centres (CHCs) in Shanghai, China, using multistage cluster sampling. An evidence-based chronic disease prevention (EBCDP) evaluation tool was employed to assess physician EBCDP awareness, adoption, implementation and maintenance based on the Reach, Effectiveness, Adoption, Implementation and Maintenance (RE-AIM) framework and using a 7-point Likert scale. Linear regression analysis was used to assess associations between each EBCDP aspect and overall EBCDP status with participant characteristics or organizational factors.

**Result:**

A total of 892 physicians from CHCs in Shanghai, China, were assessed. The physicians perceived their awareness (mean = 4.90, SD = 1.02) and maintenance (mean = 4.71, SD = 1.07) of EBCDP to be relatively low. Physicians with relatively lower job titles and monthly incomes (> 9000 RMB) tended to have relatively higher scores for the awareness, adoption, and implementation of EBCDP (*P* < 0.05). Those who had participated in one program for chronic disease prevention and control were less likely to adopt (b = − 0.284, *P* = 0.007), implement (b = − 0.292, *P* = 0.004), and maintain (b = − 0.225, *P* = 0.025) EBCDP than those who had participated in more programs. Physicians in general practice (Western medicine) had a lower level of awareness of EBCDP than those in other departments (*P* < 0.0001). Physician from CHCs located in suburban areas had lower scores for awareness (b = − 0.150, *P* = 0.047), implementation (b = − 0.171, *P* = 0.029), and maintenance (b = − 0.237, *P* = 0.002) that those from urban CHCs. Physicians in CHCs affiliated with universities had higher scores on all four EBCDP aspects that those in CHCs not affiliated with a university.

**Conclusions:**

This study provides quantitative evidence illustrating EBCDP practices among physicians in CHCs with various personal and organizational characteristics, respectively. More methods should be provided to increase the awareness of such physicians regarding EBCDP to stimulate the use of EBCDP for their patients and in connection with other public health priorities.

## Background

Epidemic chronic diseases pose significant challenges for healthcare worldwide [1]. According to NCD Countdown 2030, chronic diseases accounted for approximately 40.5 million of the 56.9 million deaths that occurred worldwide in 2016 [2]. In China too, chronic diseases, including stroke, ischemic heart disease, lung cancer, and chronic obstructive pulmonary disease (COPD), are widespread. Data indicate that stroke and ischaemic heart disease were the leading causes of death and decreased disability-adjusted life years (DALYs) at the national level in China in 2017 [3]. Against this background, the efficient global prevention and control of chronic disease has gradually become a focus for scholars [4, 5]. In China, the prevention and control of chronic diseases has been included in the Healthy China 2030 Strategic Plan. Evidence-based chronic disease prevention (EBCDP) has emerged and become widely known as a scientific tool to increase efficiency in preventing and controlling chronic disease in developed countries [6]. Evidence-based tools are utilized by health practitioners in many countries. In the US, the web portal Cancer Control P.L.A.N.E.T., which is designed to support cancer control initiatives, presents useful approaches for planning and developing evidence-based programs and policies [7–10]. In Australia, public health resource Health-Evidence.org has been the widely accepted and used by public health practitioners and the public [11]. In addition, in Australia and the US, the Cochrane Collaboration is a commonly used repository of evidence from systematic reviews [12, 13].

Currently, studies have begun to assess the extent and possible influencing factors of the implementation and dissemination of EBCDP, which is a dynamic program that involves awareness, adoption, implementation and maintenance [14]. Hannon et al. found that US cancer control practitioners had strong preferences regarding cancer control programs, but only 48% of the practitioners had used evidence-based practice resources [15]. Interviewing New York state local health department leaders, Sosnowy et al. found that although most of these individuals understood the EBP concept, relatively few had substantial expertise and experience with it [16]. Qualitative studies have shown that possible influencing factors include strong leadership, workforce capacity (i.e., numbers and skills), resources, funding and program mandates, political support, and access to data and program models suitable for community conditions [14, 16]. In recent years, studies have quantitatively revealed a possible relationship between the application of evidence-based decision-making (EBDM) and the factors influencing it, primarily based on analyses performed in the US, Australia, and Canada [17, 18]. These studies have focused on personal and organizational-level barriers to and facilitators of EBCDP. Common barriers to EBCDP include a lack of time, a lack of skills and formal training, a lack of incentives to use evidence when making decisions, a lack of funding, and an unsupportive organizational culture [19–24]. Budd et al. (2018) studied organizational and contextual factors that affect the adoption of evidence-based chronic disease interventions in the US and elsewhere [25].

Despite increasing international calls for the inclusion of EBDM in public health programming and policymaking [16, 26–29], few studies have systematically examined dynamic EBCDP practices and the factors influencing them. In lower- and middle-income countries, little quantitative information is available on the status of and factors related to these practices in primary care health institutions. For instance, in Shanghai, various intervention programs have targeted the prevention of chronic diseases, including diabetes, hypertension, and stroke, as required by public health plans in Shanghai [30]. However, there have been few studies that investigate how public health practitioners have implemented these programs and the possible factors influencing the different aspects of EBCDP.

By surveying physicians from community healthcare centres (CHCs) in Shanghai, this study quantitatively measured the implementation and dissemination of EBCDP programs in Shanghai and examined possible factors influencing the various processes involved in EBCDP from the perspective of the physician. In China, primary care health institutions are responsible for chronic disease prevention and control [10]. Therefore, we focused on CHC physicians.

## Methods

### Source

In this study, a multistage stratified cluster random sampling method was used to obtain a representative sample of physicians from CHCs in Shanghai. To make the sample comparable, a sample of CHCs was first collected randomly from 246 CHCs in Shanghai in 2019. Using a random number generator, 39 urban and 39 suburban CHCs were chosen. Then, we asked the administrations of the CHCs to assist with the survey. Ultimately, 36 CHCs in the urban area and 39 in the suburban area agreed to help, for a total of 75 CHCs. According to the average proportional distribution of various physician job titles in CHCs in Shanghai, we then randomly selected 6 junior physicians, 6 mid-level physicians, and 1 senior physician in each CHC. Finally, 975 questionnaires were provided to the participating physicians for completion between April and July 2019.

### Measurement

#### EBCDP status

To analyse the status of EBCDP and the factors influencing it in CHCs in China, we used Dreisinger et al.’s (2012) analytic framework [31–33]. According to this framework, decisions to adopt, accept and utilize an innovation result from the following dynamic process [14]: (1) awareness, which involves defining the actions taken to make target audiences aware of the innovative programs across sites and settings [32]; (2) adoption, which is the absolute number, proportion, and representativeness of institutions and practitioners who deliver a program; (3) implementation, which is the extent to which an innovation is completely executed, accounting for adaptation and costs [34]; (4) maintenance, which is the extent to which a program becomes institutionalized or part of routine organizational practices and policies [32].

We used a survey tool developed by the Prevention Research Center in St. Louis and the Missouri Foundation for Health to facilitate the dissemination of prevention interventions across the US state of Missouri [31]. All the items are measured on a 7-point Likert scale, with “1” and “7” representing “strongly disagree” to “strong agree”, respectively. We examined the reliability and validity of the evaluation tool and modified it according to the test results. The coefficient of one item (i.e., the community served by the intervention considers a certain disease to be a problem) on the overall scale was below 0.30. Therefore, this item was deleted, and 19 items remained. We found that the reliability and validity of the scale were acceptable. The Cronbach’s α of the total scale was 0.981, and the Spearman-Brown coefficient was 0.924. For the subscales, the Cronbach’s α values were 0.865, 0.959, 0.965 and 0.970, and the Spearman-Brown coefficients were 0.631, 0.950, 0.957 and 0.918 for the subscales of awareness, adoption, implementation, and maintenance, respectively. All these results indicated that the scale had satisfactory applicability for physicians from CHCs in China. The questionnaires were sent via web mail, and written informed consent was obtained from all participants.

#### Possible influencing factors

We included personal and organizational characteristics as the primary factors in this study. The personal factors were sex, age, education, position, working years, and monthly income. The organizational factors were the number of programs for chronic disease prevention and control a physician had participated in, department, CHC location (i.e., urban or suburban), and whether the CHCs were affiliated with universities. The number of programs for chronic disease prevention and control variable concerns the number of such programs the surveyed physicians had participated in. The included departments were general medicine (Western medicine), prevention and health care, general practice in traditional Chinese medicine, and other departments, such as medical technology and rehabilitation. We compared the CHC locations (urban vs. suburban) because we desired to uncover regional differences. In an urban region, there is a dense distribution of residents as well as secondary and tertiary hospitals. Thus, in such regions, there may be more investment in training programs or chronic disease prevention programs involving EBCDP for physicians. In a suburban region, because there are fewer secondary and tertiary hospitals, more efforts are devoted to the provision of medical healthcare by physicians instead of chronic disease prevention and control [35]. EBCDP practice may be impacted by this factor. Regarding the CHC-university affiliation variable, CHC affiliation with a university indicates the availability of more evidence-based resources, including literature resources and training from researchers at these universities [36].

### Statistical analysis

All statistics were performed using SAS 9.0. Descriptive analysis was used to describe participant characteristics and the factors that possibly influenced EBCDP practice. T-tests and ANOVA were used to analyse the different factors affecting the various EBCDP aspects, including awareness, adoption, implementation, maintenance, and overall EBCDP status. Finally, we used linear regression to examine the relationship between independent variables, including the personal and organizational factors and the dependent variables of each EBCDP aspect (awareness, adoption, implementation and maintenance) and overall EBCDP status.

## Results

### Participant demographics

Table 1 provides descriptions of the physicians and CHCs investigated in this study. A total of 892 valid questionnaires were collected from 75 CHCs. The effective response rate was 91.49%. The percentages of males (49.10%) and females (50.90%) were similar. The largest proportion of physicians was in the 31–40 year age group (46.97%). The majority of the participants had bachelor’s degrees (77.69%). Most of the physicians held junior (46.63%) and mid-level (45.74%) titles and had worked for ≤5 years (36.38%). The largest proportion (43.16%) earned monthly incomes of 6001–9000 RMB. Regarding the number of chronic disease prevention and control programs, 33.63% of the physicians had participated in 1 program, and 19.73% had taken part in ≥5 programs. Concerning departmental affiliation, most physicians were in general medicine (Western medicine) (53.59%). More physicians were from CHCs located in urban areas (54.26%), and a small proportion was from CHCs affiliated with a university (21.30%).

**Table 1:** Demographics and participant perceptions of the various aspects of EBCDP (*n* = 892)

Variable	Classification	n	%
Sex	Male	438	49.10
	Female	454	50.90
Age (year)	≤30	194	21.75
	31–40	419	46.97
	41–50	240	26.91
	> 50	39	4.37
Education	Associate’s degree or below	89	9.98
	Bachelor’s degree	693	77.69
	Master’s degree or higher	110	12.33
Position	Junior	416	46.63
	Mid-level	408	45.74
	Senior	68	7.62
Working years (years)	≤5	322	36.38
	6–10	232	26.21
	11–15	153	17.29
	> 15	178	20.11
Income (RMB, Ұ)	≤6000	344	38.57
	6001-9000	385	43.16
	> 9000	163	18.27
Number of chronic disease prevention and control programs	1	300	33.63
	2	131	14.69
	3	155	17.38
	4	130	14.57
	≥5	176	19.73
Department	General medicine (Western medicine)	478	53.59
	Prevention and health care	228	25.56
	General practice (Chinese medicine)	91	10.20
	Other departments	95	10.65
Area	Urban	484	54.26
	Suburban	408	45.74
Affiliated with a university	Yes	190	21.30
	No	702	78.70
Awareness (X ± S)		4.902	1.022
Adoption (X ± S)		5.048	1.097
Implementation (X ± S)		4.995	1.073
Maintenance (X ± S)		4.711	1.066
Overall EBCDP status (X ± S)		4.869	0.998

The CHC physicians scored lowest on the perception of their maintenance of EBCDP (mean = 4.71, SD = 1.07), representing slightly agree. The score for EBCDP awareness was also relatively low (mean = 4.90, SD = 1.02). Comparatively, the scores for adoption (mean = 5.05, SD = 1.10, representing moderately agree) and implementation (mean = 5.00, SD = 1.07, representing moderately agree) were higher. The overall EBCDP score was 4.869, representing slightly agree.

### Effects of physician characteristics on EBCDP processes

Table 2 shows the distribution of the scores for the EBCDP aspects stratified according to physician characteristics. Regarding the sex and age groups, there were no significant differences between the subgroups in their scores for the various EBCDP aspects. Concerning education, physicians with an associate’s degree or lower were more likely to have a relatively higher score for maintenance (*P* = 0.046). Regarding the level of physician, compared with mid-level and senior physicians, junior physicians had higher scores for awareness (*P* = 0.018), adoption (*P* = 0.044), and implementation (*P* = 0.016). Regarding the number of programs, the results indicated that those who participated in one program had significantly lower scores for adoption than those who participated in more programs (mean = 4.939, *P* = 0.042). In addition, there were no significant differences between subgroups for the variables working years and income.

**Table 2:** Possible factors affecting EBCDP awareness, adoption, implementation and maintenance by physicians

Variable	Classification	X	Awareness				Adoption				Implementation				Maintenance				Overall		
			S	F/t value	***P*** value	X	S	F/t value	***P*** value	X	S	F/t value	***P*** value	X	S	F/t value	***P*** value	X	S	F/t value	***P*** value
Sex	Male	4.871	1.002	−0.890	0.372	5.016	1.107	− 0.850	0.395	4.967	1.086	−0.760	0.445	4.693	1.086	−0.500	0.615	4.844	1.011	−0.730	0.463
	Female	4.932	1.041			5.079	1.088			5.022	1.061			4.729	1.047			4.893	0.986		
Age (years)																		4.869	0.998		
	≤30	5.029	1.002	1.600	0.189	5.108	1.037	0.360	0.779	5.105	1.027	1.190	0.311	4.867	0.984	2.210	0.086	4.993	0.942	1.640	0.178
	31–40	4.853	1.007			5.021	1.103			4.956	1.065			4.649	1.053			4.821	0.984		
	41–50	4.865	1.049			5.033	1.133			4.950	1.104			4.670	1.111			4.832	1.041		
	> 50	5.017	1.079			5.128	1.120			5.128	1.182			4.849	1.248			4.993	1.119		
Education	Associate’s degree or lower	5.022	1.134	1.420	0.241	5.221	1.235	1.880	0.153	5.189	1.235	2.720	0.066	4.906	1.216	3.080	0.046	5.048	1.152	2.840	0.059
	Bachelor’s degree	4.906	1.005			5.046	1.089			4.996	1.068			4.714	1.048			4.871	0.988		
	Master’s degree or higher	4.779	1.029			4.918	1.020			4.833	0.942			4.531	1.028			4.711	0.906		
Title	Junior	4.986	0.974	4.010	0.018	5.127	1.069	3.130	0.044	5.074	1.051	4.190	0.016	4.784	1.044	2.380	0.093	4.946	0.965	3.580	0.028
	Mid-level	4.859	1.025			5.008	1.083			4.967	1.058			4.668	1.034			4.830	0.983		
	Senior	4.642	1.222			4.799	1.299			4.682	1.237			4.522	1.334			4.627	1.223		
Working years (years)	≤5	4.980	0.980	1.450	0.226	5.113	1.084	0.780	0.504	5.078	1.065	1.990	0.114	4.771	1.055	0.960	0.413	4.939	0.979	1.380	0.248
	6–10	4.819	1.067			4.981	1.072			4.891	1.071			4.656	1.037			4.795	0.987		
	11–15	4.924	1.013			5.059	1.117			5.064	1.064			4.753	1.065			4.910	0.998		
	> 15	4.830	1.051			4.998	1.152			4.906	1.096			4.631	1.132			4.793	1.053		
Income (RMB, Ұ)	≤ 6000	4.873	1.045	1.690	0.184	5.011	1.086	1.300	0.274	4.942	1.080	0.980	0.376	4.710	1.098	0.290	0.745	4.844	1.016	0.760	0.469
	6001-9000	4.871	1.038			5.029	1.136			5.006	1.092			4.689	1.068			4.855	1.012		
	> 9000	5.035	0.923			5.172	1.023			5.082	1.013			4.765	0.992			4.955	0.924		
Number of chronic disease prevention and control programs	1	4.846	1.045	0.840	0.499	4.939	1.111	2.490	0.042	4.870	1.107	2.220	0.065	4.586	1.068	2.080	0.081	4.758	1.011	2.080	0.082
	2	4.883	0.867			5.127	1.023			5.026	0.974			4.827	0.925			4.936	0.879		
	3	4.903	1.044			4.955	1.084			4.995	1.074			4.689	1.043			4.845	0.991		
	4	4.887	1.081			5.079	1.099			5.018	1.109			4.737	1.161			4.889	1.053		
	≥5	5.021	1.024			5.233	1.118			5.168	1.041			4.837	1.092			5.016	1.011		
Department	General medicine (Western medicine)	4.816	0.985	6.830	0.0001	4.932	1.066	7.520	<.0001	4.865	1.005	8.310	<.0001	4.567	1.007	10.310	<.0001	4.742	0.940	9.870	<.0001
	Prevention care	4.924	0.995			5.077	1.064			5.092	1.123			4.808	1.108			4.944	1.023		
	General medicine (Traditional Chinese medicine)	4.853	1.122			5.110	1.211			4.985	1.140			4.721	1.017			4.873	1.034		
	Other departments	5.326	1.074			5.502	1.103			5.427	1.097			5.192	1.141			5.324	1.048		
Area	Urban	4.997	1.012	3.060	0.002	5.169	1.098	3.610	0.0003	5.124	1.058	3.940	<.0001	4.867	1.025	4.810	<.0001	5.003	0.980	4.400	<.0001
	Suburb	4.788	1.023			4.904	1.079			4.842	1.072			4.526	1.085			4.710	0.997		
Affiliated with a university	Yes	5.054	1.093	2.330	0.020	5.281	1.131	3.320	0.001	5.220	1.096	3.270	0.001	5.022	1.087	4.580	<.0001	5.120	1.033	3.940	<.0001
	No	4.860	0.998			4.985	1.080			4.934	1.059			4.627	1.045			4.801	0.978		

The organizational factors displayed stronger effects. Regarding the department, physicians from general medicine (Western medicine) had the lowest scores for awareness (mean = 4.816, *P* = 0.0001), adoption (mean = 4.932, *P* < 0.0001), implementation (mean = 4.865, *P* < 0.0001), and maintenance (mean = 4.567, *P* < 0.0001). In terms of CHC location, in contrast to physicians at suburban CHCs, physicians at urban CHCs had higher scores on all four of the aspects: 4.997 vs. 4.788 (urban VS suburb, *P* = 0.002) for awareness, 5.169 vs 4.904 (*P* = 0.0003) for adoption, 5.124 vs. 4.842 (*P* < 0.0001) for implementation, and 4.867 vs. 4.526 (*P* < 0.0001) for maintenance. Last, the results indicated that physicians in university-affiliated CHCs had relatively higher scores for awareness (*P* = 0.020), adoption (*P* = 0.001), implementation (*P* = 0.001), and maintenance (*P* < 0.0001) than physicians at CHCs not affiliated with a university.

Regression analysis of EBCDP aspects and personal and organizational factors.

Linear regression was used to analyse the differences within the four EBCDP aspects based on various personal and organizational factors (Table 3). The results indicate that compared with the group aged ≤30 years, physicians aged 31–40 years were less likely to maintain EBCDP (b = − 0.218, *P* = 0.036). There were no significant differences based on education level or years of work. Interestingly, lower level physicians had higher scores for the awareness, adoption, and implementation of EBCDP (*P* < 0.05). Compared with those who had a monthly income of 9000 RMB per month, physicians with a monthly income ≤6000 RMB (awareness: b = − 0.255, *P* = 0.011; adoption: b = − 0.217, *P* = 0.042; implementation: b = − 0.229, *P* = 0.027) and 6001–9000 RMB (awareness: b = − 0.247, *P* = 0.011; adoption: b = − 0.204, *P* = 0.049; overall: b = − 0.187, b = 0.045) had lower scores. Compared with physicians who participated in more than five programs, those who participated in one program had lower scores for adoption (b = − 0.284, *P* = 0.007), implementation (b = − 0.292, *P* = 0.004), maintenance (b = − 0.225, *P* = 0.025) and overall EBCDP status (b = − 0.244, *P* = 0.010).

**Table 3:** Regression analysis of differences within various EBCDP aspects

Variable	Classification		Awareness		Adoption		Implementation		Maintenance		Overall	
			B	*P* value	B	*P* value	B	*P* value	B	*P* value	B	*P* value
Sex	Male	Reference										
	Female		0.029	0.670	0.029	0.695	0.021	0.770	0.003	0.963	0.016	0.809
Age (years)	≤30	Reference										
	31–40		−0.123	0.225	−0.034	0.755	−0.114	0.278	−0.218	0.036	−0.147	0.134
	41–50		−0.051	0.695	0.051	0.714	−0.051	0.710	−0.132	0.325	−0.069	0.584
	> 50		0.185	0.376	0.223	0.320	0.205	0.347	0.103	0.630	0.162	0.422
Education	Associate’s degree or lower	Reference										
	Bachelor’s degree		0.052	0.691	−0.006	0.965	−0.009	0.949	0.038	0.777	0.021	0.868
	Master’s degree or higher		−0.122	0.469	−0.180	0.320	−0.227	0.196	−0.178	0.306	−0.182	0.263
Title	Senior	Reference										
	Junior		0.466	0.026	0.545	0.015	0.468	0.031	0.321	0.134	0.418	0.038
	Mid-level		0.323	0.043	0.345	0.043	0.394	0.018	0.199	0.224	0.293	0.057
Working years (year)	≤5	Reference										
	6–10		−0.122	0.347	− 0.101	0.466	− 0.221	0.101	− 0.091	0.493	− 0.131	0.291
	11–15		0.013	0.939	0.012	0.948	−0.041	0.814	0.057	0.742	0.017	0.917
	> 15		0.031	0.869	0.088	0.665	−0.054	0.784	0.020	0.918	0.013	0.943
Income (RMB, Ұ)	> 9000	Reference										
	≤6000		−0.255	0.011	−0.217	0.042	−0.229	0.027	−0.146	0.154	−0.196	0.041
	6001–9000		−0.247	0.011	−0.204	0.049	−0.159	0.113	−0.175	0.078	−0.187	0.045
Number of programs	≥5	Reference										
	1		−0.173	0.079	−0.284	0.007	−0.292	0.004	−0.225	0.025	−0.244	0.010
	2		−0.102	0.387	−0.067	0.597	−0.107	0.383	0.046	0.706	−0.036	0.754
	3		−0.081	0.473	−0.237	0.050	−0.132	0.261	−0.099	0.392	−0.127	0.244
	4		−0.103	0.387	−0.112	0.375	−0.104	0.398	−0.041	0.739	−0.078	0.492
Department	General medicine (Western medicine)	Reference										
	Preventive care		0.113	0.191	0.138	0.134	0.229	0.011	0.217	0.014	0.191	0.021
	General medicine (Traditional Chinese medicine)		0.066	0.576	0.217	0.086	0.161	0.190	0.175	0.148	0.161	0.157
	Other departments		0.505	<.0001	0.562	<.0001	0.551	<.0001	0.588	<.0001	0.561	<.0001
Area	Urban	Reference										
	Suburban		−0.150	0.047	−0.148	0.066	−0.171	0.029	−0.237	0.002	−0.192	0.008
Affiliated with a university	Yes	Reference										
	No		−0.156	0.077	−0.241	0.011	−0.224	0.015	−0.321	0.0001	−0.257	0.003

In terms of department, interestingly, we found that compared with general practice (Western medicine) physicians, those in other departments had higher scores for awareness (b = 0.505, *P* < 0.0001), adoption, implementation, maintenance and overall (*P* < 0.0001). Concerning the areas in which the CHCs were located, compared with urban CHC physicians, physicians from suburban CHCs had lower scores for awareness (b = − 0.150, *P* = 0.047), implementation (b = − 0.171, *P* = 0.029), maintenance (b = − 0.237, *P* = 0.002), and overall (b = − 0.192, *P* = 0.008). Physicians in CHCs affiliated with a university had higher scores for adoption (b = − 0.241, *P* = 0.011), implementation (b = − 0.224, *P* = 0.015), maintenance (b = − 0.321, *P* = 0.0001) and overall (b = − 0.257, *P* = 0.003).

## Discussion

Over the past several years, many efforts have been devoted to developing EBDM and promoting its implementation among public health practitioners in developed countries. It has been found that among US and European public health practitioners, 56–64% of chronic disease prevention interventions currently in use are evidence-based [37], while quantitative estimates of EBCDP use and possible factors influencing that use in lower- and middle-income countries are rare [38]. In this study, we evaluated physician EBCDP practice and possible personal and organizational factors affecting that practice in Shanghai, China.

The results indicate that physicians perceived their EBCDP adoption and implementation to be strong. However, they had relatively lower levels of awareness and maintenance with respect to EBCDP. This outcome is in accordance with Shi et al.’s (2019) qualitative study in China, which found that physicians integrate evidence into chronic disease prevention practices poorly [39], as suggested by the lack of a well-developed evidence base and low levels of physician awareness, adoption, implementation, and adequate maintenance of EBCDP; similar results have been observed in other Asian and developing countries [40, 41]. For instance, Jirawattanapisal et al. compared healthcare data collection, sharing, and use in Thailand, Mainland China, South Korea, Taiwan, Japan, and Malaysia and found that many data are not used. One can speculate that obtaining persuasive evidence and data accessibility are important issues to be addressed in developing countries [41]. Realizing the importance of using evidence, China has gradually implemented evidence-based approaches to target several common chronic diseases, in part by making data more available. The Health Ministry has issued a series of technical guidance manuals for the prevention and treatment of diseases in CHCs, including cardiovascular diseases, type II diabetes, chronic hepatitis B, and tumours [42, 43]. Additionally, we found that the maintenance of EBCDP, that is, the extent to which a program becomes institutionalized as part of routine organizational practices and policies, was not a particular problem in China, unlike in other developing and developed countries [21, 22].

Concerning the specific factors that influence EBCDP practice, we analysed the effect of physician demographics. First, physicians who were younger and held junior titles were more likely to practice EBCDP. This finding may be explained by the availability of evidence-based concepts and training courses, as this population may have had more exposure to EBCDP. Additionally, these groups of physicians were relatively new to their work and typically more likely than their colleagues with longer tenure to acquire new knowledge and adopt new methods. Second, we found that those who participated in only one chronic disease prevention and control program were less inclined to adopt, implement and maintain EBCDP, reflecting that the programs provided by the government can directly increase physician consciousness and practice of EBCDP. This finding is consistent with Hannon’s (2010) study that found that program participation could successfully change practitioner behaviour [15].

Regarding organizational barriers, first, it was interesting to note that general practitioners (GPs) had lower levels of awareness of EBCDP than other physicians. In China, GP training differs from that of public healthcare physicians and physicians working in medical technology and rehabilitation departments. Therefore, the standardized and on-the-job training courses for GPs should include more evidence-based elements in China. Additionally, in China, it is common that although GPs are required to assume responsibility for chronic disease prevention and control, many GPs focus more on disease treatment [44]. Second, in this study, physicians from CHCs in urban areas had greater awareness of EBCDP than those from CHCs in suburban areas. This finding may be influenced by organizational management practices and policy factors. Budd et al. confirmed that organizational culture greatly influences EBCDP [25]. In addition, EBCDP practice was strongly influenced by the incentive policy of the local health government. In urban areas, there may be more investment in training programs or chronic disease prevention programs involving EBCDP [25]. Third, the results indicated that if a CHC is affiliated with a university, its physicians might be able to access more evidence-based resources and have greater opportunity to collaborate with researchers. Therefore, their awareness of EBCDP is greater. In China, particularly in large cities, many CHCs have established cooperative relationships with universities with regard to academic and clinical issues. This finding also suggests that to increase physician EBCDP practice, more training should be provided. In the US, EBDM training courses have been conducted in both Kansas and Mississippi to address gaps in competencies among public health practitioners. Evidence-based public health training has been found to be an effective method of integrating new knowledge and skills into the public health workforce [37, 45]. Such training can include programs focused on specific EBDM skills or on incentives and policies that could affect the organizational culture in a workplace [46].

### Limitations

This study has several limitations. First, the analysed data were self-reported, which may result in bias due to the ability of a participant to recall and/or report the requisite information. Survey respondents were provided a standard definition of EBDM before completing the questionnaire. However, the results should still be interpreted with caution given that they were self-reported. Second, regarding the study design, although the sample CHCs were randomly chosen in the cross-sectional study, the participant sample was not well randomized. Participants were selected at each CHC mainly with the assistance of administrators. As a result, the age of our sample was relatively young. Third, because the sample was chosen from CHCs in Shanghai, the survey may not be representative of other cities. Fourth, since the study focused on personal and organizational-level barriers, additional research on the political and sociocultural barriers that influence EBCDP is required.

## Conclusion

This study provides robust quantitative evidence regarding the EBCDP practice of physicians from CHCs with various personal and organizational characteristics, respectively. The CHC physicians perceived their awareness and maintenance of EBCDP to be relatively low. Those who had participated in fewer chronic disease prevention and control programs, worked in general practice (Western medicine), were from suburban CHCs, and were at CHCs not affiliated with universities exhibited lower EBCDP-related outcomes. More methods should be developed to improve these participants’ EBCDP practice and to stimulate the use of EBCDP for chronic disease prevention and other public health priorities. This study represents an important step in this direction and identifies several potential avenues for future research.

## Data Availability

The data set supporting the conclusions of this article is available from the corresponding author on reasonable request.

## Abbreviations

EBDM: Evidence-based decision-making
CHCs: Community healthcare centre
EBCDP: Evidence-based chronic disease prevention
